# Viewpoint: Optimising Cancer Treatment to Reduce Its Environmental Impact

**DOI:** 10.1111/1754-9485.70052

**Published:** 2025-12-02

**Authors:** Robert Chuter, Kari Tanderup

**Affiliations:** ^1^ Christie Medical Physics and Engineering (CMPE) The Christie NHS Foundation Trust Manchester UK; ^2^ Division of Cancer Sciences, School of Medical Sciences, Faculty of Biology, Medicine and Health University of Manchester Manchester UK; ^3^ Danish Center for Particle Therapy Aarhus University Hospital Aarhus Denmark; ^4^ Department of Clinical Medicine Aarhus University Aarhus Denmark

**Keywords:** clinical and cost benefit, physics, politics (medical/radiological), radiation oncology

## Abstract

It is widely accepted that treatment and care for patients with cancer must and should happen, as it is what everyone would want for themselves and their loved ones. Everyone wants the best possible care, but climate change and the extreme weather that it causes are increasingly affecting everyone including patients in negative ways. To continue giving the best possible cancer care without harming our environment and therefore patients, there is a need to optimise care. Through improving efficiency, reducing travel, using renewable energy and other measures it is possible to limit our environmental impact whilst still giving the best care available. In many cases this creates a win‐win‐win scenario for patients and the environment and, in many cases, significant cost savings.

1

Giving the best possible cancer treatments and care to patients is not only our job, but also what we would each want for ourselves. However, climate change and associated extreme weather are increasingly affecting us and our patients [1, 2]. For example, Australia is likely to see an increase in the frequency and intensity of bushfires, floods [3, 4] and more extreme heat [5] (see Figure 1). Conservative estimates have equated the carbon footprint responsible for one death at about 4000 tons of carbon dioxide equivalent (tCO_2_e), highlighting the direct link between health and climate change [7, 8]. Radiotherapy (RT) is used to treat 50% of all cancer patients [9]. In most RT, the tumours are treated with high‐energy radiation produced by linear accelerators (linacs). Establishing RT facilities with linear accelerators involves a significant carbon footprint due to the construction of buildings, radiation shielding [10] and the production of the 5‐to‐10‐ton linear accelerators which are equipped with advanced electronics. In addition to this, most radiotherapy is delivered over many visits, typically between 15 and 30 separate visits per patient. These, combined with the increased use of high‐specification computers and energy‐hungry technology, means that radiotherapy is likely to have a high carbon footprint. Studies have shown that patient travel to and from treatment is the largest contributor [11, 12]. A comprehensive life cycle assessment (LCA) of radiotherapy in the USA put the carbon footprint of a 25‐fraction course at 4310 kgCO_2_e [13].

**FIGURE 1:**
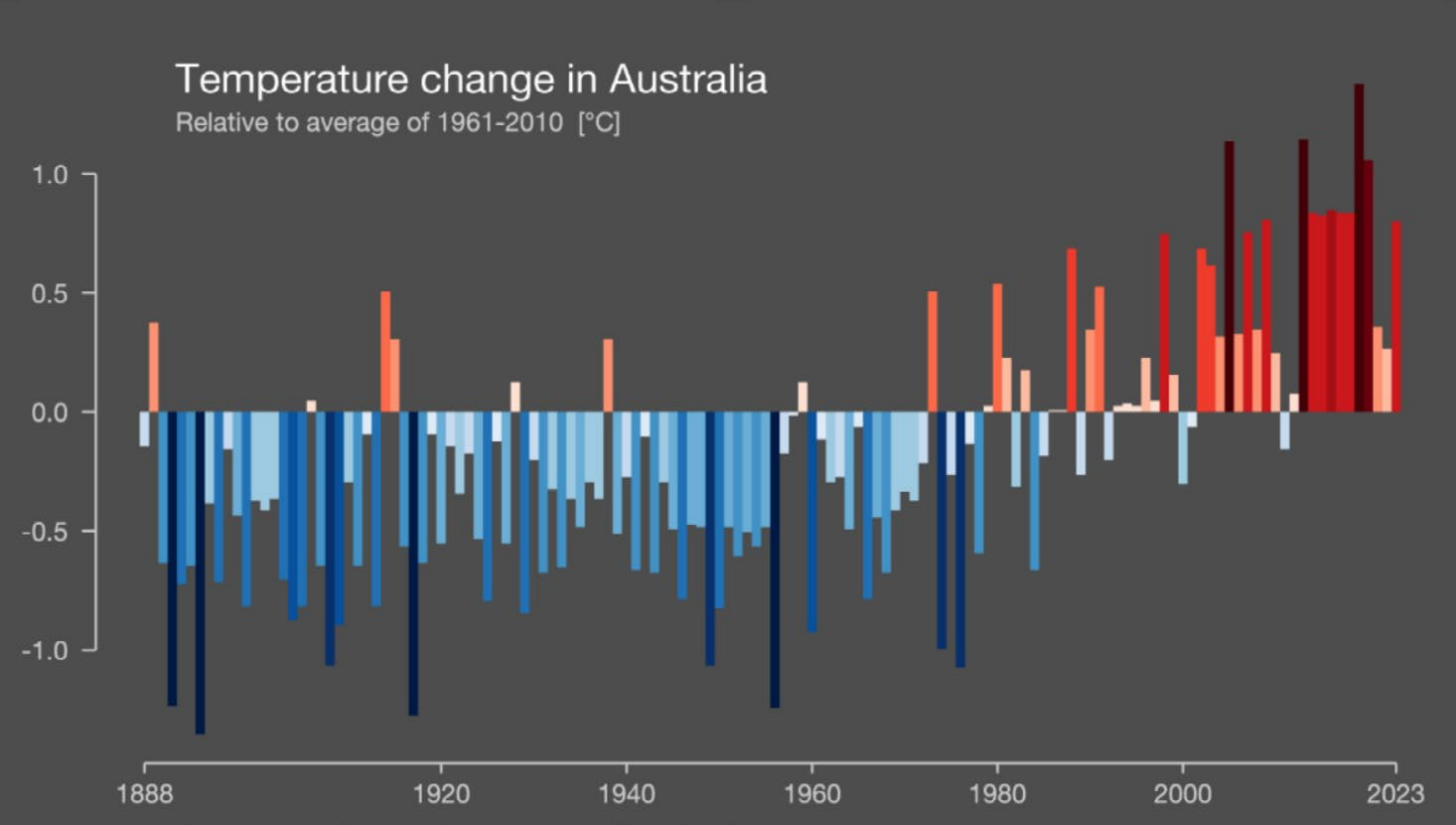
Bar chart showing the temperature change in degrees Celsius in Australia relative to the mean temperature between 1961 and 2010 [6]. A blue bar indicates a temperature below the 1961–2010 average and a red bar indicates a temperature above that average; the darker the colour the further from the average the value is.

This certainly does not mean that we should stop treating people with cancer, but it should motivate us to optimise cancer treatments, improve efficiency and reduce waste so that we give the best treatment to patients whilst also minimally affecting the environment that we all share. It may also save money as using less resources tends to cost less. There are many ways that we could offer treatments with a lower carbon footprint but comparable clinical outcomes. For example, a recent LCA showed that using SABR instead of surgery to treat early‐stage NSCLC reduced the carbon footprint by 68% [14]. Another approach would be to reduce patient travel.

Recent international clinical trials have shown that shorter courses of RT (delivered over 3–5 visits) are as effective as standard courses (15–30 visits) in terms of tumour control and toxicity in several patient groups. This approach, called hypofractionation, has been shown to be as effective as standard treatments for three of the most common cancers: early‐stage breast cancer [15, 16], localised prostate cancer [17, 18] and early‐stage non‐small cell lung cancer [19, 20]. Increased use of hypofractionation could therefore significantly reduce the carbon footprint of radiotherapy by reducing the number of visits the patient needs to make.

Hypofractionated RT is more complex to plan due to higher dose per fraction, which requires increased accuracy of treatment delivery. This makes it more complex to deliver because it requires more imaging before each treatment and thus increases the use of motion management techniques compared to standard treatments, potentially reducing any reduction in carbon footprint [21].

Additionally, there is a large regional variability in the uptake of hypofractionated RT [22]. There is evidence that some patients seek out hypofractionated RT at centres further away than their local centre, where this is not an option [23]. In turn, this can increase the total distance that patients travel. However, if hypofractionated RT were made the standard and implemented wherever it is clinically acceptable, this would no longer be an issue.

Hypofractionation has the potential to be used in many more cancer indications than those where efficacy and safety have already been demonstrated. There is therefore an urgent need to prioritise and fund clinical trials which aim to prove efficacy, efficiency and safety of hypofractionated radiotherapy. Funding agencies need to prioritise and promote clinical trials which have potential for carbon footprint reductions.

In addition to reducing the environmental impact of cancer treatment [24], reducing patient travel by adopting hypofractionation or a satellite centre is likely to also improve patient experience due to the decreased burden of treatment. It may also reduce the pressure on busy departments by reducing the time spent on the linac overall.

The proportion of patients using public transport to travel to and from RT is not well understood but from preliminary work undertaken in the UK it is likely to be about 30% for treatment centres not well connected to public transport. This proportion is likely to be lower in Australia due to its low population density and large distances between cities. The low public transport use is likely driven by side effects, which make travelling by public transport uncomfortable. So, it is plausible that, as short‐term side effects commonly take a week or two to start, hypofractionated RT may also increase the use of public transport since it is less burdensome and less uncomfortable. Increasing the use of public transport as well as potentially building more, smaller radiotherapy centres (which can sometimes be satellite centres) [25] and enabling patients to travel from further afield by staying in local accommodation, are other initiatives that should be looked at. Ensuring that a patient's appointments are optimised across their care pathway is also key, for example by ensuring that chemotherapy and RT appointments happen close together, both geographically and in time.

There are other parts of the RT workflow that require attention such as the re‐use of immobilisation equipment, improved energy efficiency of the linacs, de‐carbonisation of the electricity grid and heating systems, reducing the use of SF_6_ gas—which has a very large global warming potential—and de‐carbonising staff travel [26] either through public or active travel and—where necessary—electric cars [11, 27]. Additionally, the amount of data that we store is vastly increasing through restrictions on and caution with deletion, as well as through the increased use of image guided RT, including MR guided RT, meaning more image data is being stored. Creating the images themselves as well as the computers used to power them and process the images use energy, but it is possible that the servers used to store the data have more impact [12]. Optimising how much data is kept, which is often governed by a country's regulations, should be examined [11]. Some of these changes would require modifying regulations, potentially requiring lobbying to make these happen.

Lack of knowledge and awareness about environmental sustainability in radiation oncology is a barrier to the implementation of environmentally sustainable strategies. Research in the carbon footprint of radiotherapy is an emerging field. Over the past 2 years, the first publications outlining results on the carbon footprint of linac‐based radiotherapy were published. In parallel, the first initiatives to implement some of this knowledge into centres have started in the UK [28, 29] and Canada [30]. These initiatives aim to summarise what has been learnt and make it easier for interested and motivated staff to make changes in their centres. The carbon footprint of other radiotherapy modalities, such as proton therapy and brachytherapy, is still being explored. Even though, it is likely that both proton therapy and MR‐guided RT have significantly larger carbon footprints than standard linac‐based radiotherapy based on some initial work [31, 32].

As the entire research field is new, the radiotherapy community is not yet systematically knowledgeable about the environmental impact of radiotherapy and the most important mitigation strategies for improved radiotherapy sustainability. The topic of environmental sustainability is also not systematically embedded in curricula relevant for radiation oncology, and there is a significant potential to improve dissemination and education, through this and other initiatives.

In 2024, the European Society of Radiotherapy and Oncology (ESTRO) agreed on strategic principles to prioritise environmental sustainability, having already formed a ‘Green Task Force’ in 2022. In this context, ESTRO aims to help increase awareness and facilitate the dissemination of research within the field of environmental sustainability of radiotherapy. Over the last 2–3 years, several initiatives were launched including sessions on sustainability at the ESTRO annual conferences, campaigns for sustainable travelling, webinars, online teaching/meetings/activities to reduce travelling [33, 34], as well as the new section ‘Society Matters – Sustainability, Education and Health Care Policy’ in the ESTRO Green Journal [35]. Furthermore, ESTRO is currently developing a new digital platform, which will improve online accessibility for contents and events and thereby decrease carbon emissions from travelling—which is particularly important for long‐haul travel such as inter‐continental flying from Australia. ESTRO can still do more, and other national and international societies and organisations need to step up their commitments.

There are many hurdles to implementing these changes, including hypofractionation and other potential changes more widely, but the threat of climate change is arguably greater than the threat COVID‐19 posed in 2020. To combat COVID‐19 the community swiftly implemented new protocols, including hypofractionation to reduce footfall [36]. It is now time that we rapidly change practice as widely as possible to address the climate crisis as well.

## Author Contributions

**Robert Chuter:** conceptualization, methodology, writing – original draft, writing – review and editing. **Kari Tanderup:** conceptualization, methodology, writing – original draft, writing – review and editing.

## Funding

This work was supported by Health Services and Delivery Research Programme, NIHR155944.

## Conflicts of Interest

The authors declare no conflicts of interest.

## Data Availability

Data sharing not applicable to this article as no datasets were generated or analysed during the current study.
